# Electrodynamic study of YIG filters and resonators

**DOI:** 10.1038/srep34739

**Published:** 2016-10-04

**Authors:** Jerzy Krupka, Bartlomiej Salski, Pawel Kopyt, Wojciech Gwarek

**Affiliations:** 1 Institute of Microelectronics and Optoelectronics, Warsaw University of Technology, Warsaw, 00662, Poland; 2 Institute of Radioelectronics and Multimedia Technology, Warsaw University of Technology, Warsaw, 00665, Poland

## Abstract

Numerical solutions of coupled Maxwell and Landau-Lifshitz-Gilbert equations for a magnetized yttrium iron garnet (YIG) sphere acting as a one-stage filter are presented. The filter is analysed using finite-difference time-domain technique. Contrary to the state of the art, the study shows that the maximum electromagnetic power transmission through the YIG filter occurs at the frequency of the magnetic plasmon resonance with the effective permeability of the gyromagnetic medium *μ*_*r*_ ≈ −2, and not at a ferromagnetic resonance frequency. Such a new understanding of the YIG filter operation, makes it one of the most commonly used single-negative plasmonic metamaterials. The frequency of maximum transmission is also found to weakly depend on the size of the YIG sphere. An analytic electromagnetic analysis of resonances in a YIG sphere is performed for circularly polarized electromagnetic fields. The YIG sphere is situated in a free space and in a large spherical cavity. The study demonstrates that both volume resonances and magnetic plasmon resonances can be solutions of the same transcendental equations.

Yttrium iron garnet (YIG) is one of the most frequently used magnetic materials for constructing resonance and non-resonance devices operating at microwave frequencies and it is very important for ultrafast and ultrahigh-density spintronics. A theory for the ferromagnetic resonance, spin waves and modes of operation of spherical YIG resonators and filters was developed over 60 years ago^1^,^2^,^3^,^4^,^5^,^6^,^7^,^8^,^9^,^10^,^11^,^12^,^13^,^14^,^15^,^16^,^17^,^18^,^19^,^20^,^21^ and is summarized in textbooks^11^,^22^. The ferromagnetic resonance phenomenon is quantitatively described by a permeability tensor that can be derived from the Landau-Lifshitz-Gilbert equations. If a uniform static magnetic field is applied along the *z*-axis of a cylindrical or Cartesian coordinate system, then the permeability tensor in these systems takes the following form^11^:


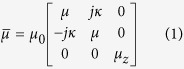



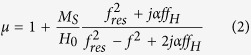



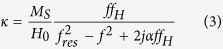



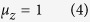


where: *f*_*H*_ = *γH*_*0*_, *f*_*res*_ = *f*_*H*_(1 + *α*^2^)^0.5^, *M*_*S*_ is the saturation magnetization of the ferromagnetic sample, *α* is the Gilbert damping factor, *H*_0_ is the static magnetic field inside the sample, γ = 35.176 MHz/(kA/m), and *j* is the imaginary unit.

Equations (2, 3, 4) are valid for *H*_*0*_ > *M*_*S*_. For frequencies *f* close to *f*_*res*_, the permeability tensor components *μ* and *κ* have Lorentzian frequency dependence. When *f* = *f*_*res*_, the imaginary parts of these components exhibit maxima and the real parts change their signs from positive to negative. Existing theories of spherical YIG band-pass filters are usually based on simple lumped-element models^9^. Such models describe the tuning of the filter, assuming that its centre frequency follows the frequency of ferromagnetic resonance but they do not explain the physical phenomenon based on electrodynamics. There remain open questions related to the operation of YIG filters and resonators. For example, does the maximum transmittance of the filter occur at the ferromagnetic resonance frequency, *f* = *f*_*res*_, where magnetic losses of YIG are the largest? Is the magnetic field uniform in the YIG sample at the frequency of maximum transmittance as predicted by magneto-quasistatic models^5^,^6^? How is the maximum transmittance of the filter related to the permeability of the YIG sphere? The goal of this work is to investigate these questions using a rigorous electromagnetic analysis.

We first numerically analysed a one-stage spherical YIG filter using the finite-difference time-domain (FDTD) method implemented in the QuickWave 3D (QW-3D) simulator^23^. This simulation uses the gyrotropic permeability tensor, as in Eqs (1–4)^24^. In addition, our basic findings were confirmed using the finite element method (FEM) implemented in the HFSS^25^ simulator. Second, we analysed a rigorous electromagnetic model of the resonances in gyromagnetic spheres situated in a free space and in spherical metal cavities.

## Analysis of a YIG filter

**Figure 1:**
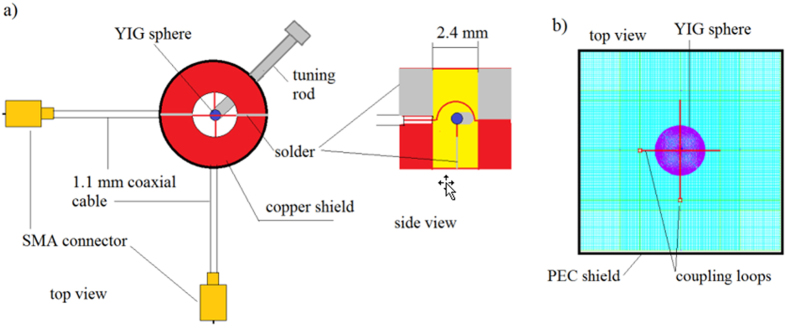
YIG filter. (**a**) Experimental set-up for a one-stage spherical YIG filter. (**b**) FDTD model of the filter.

We performed an electromagnetic analysis for the experimental setup of a one-stage filter, as shown in Fig. 1a.

**Figure 2:**
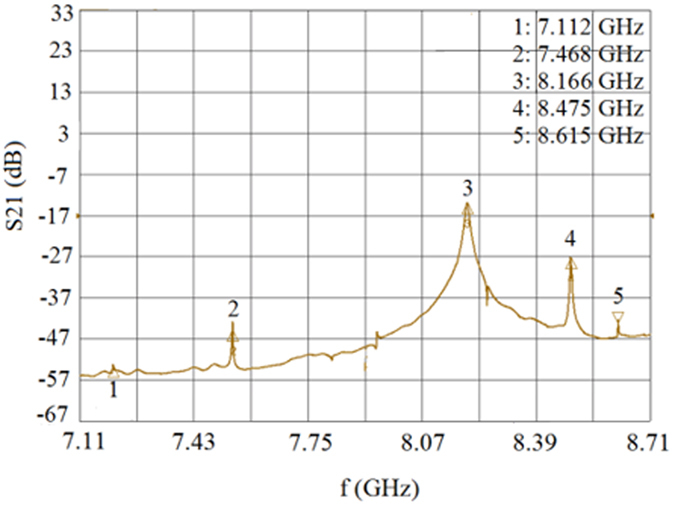
YIG filter transmission spectrum. Magnitude of a transmission coefficient |S_21_| for the one-stage spherical YIG filter measured for the [100] YIG sphere orientation and an external static magnetic field *H*_*ext*_  ≈ 222 kA/m.

In the experiment, a YIG sphere is mounted on the top of a dielectric tuning rod and inserted into a copper shield containing two orthogonal semi-circular loops. Figure 2 shows a typical transmittance of the filter at the [100] orientation of the YIG sphere (hard magnetization orientation) and at an external static magnetic field *H*_*ext*_ ≈ 222 kA/m. The radius of the YIG sphere is *R*_*1*_ = 253 μm. In the experiment, all resonance frequencies varied when we rotated the tuning rod (and the sphere) as this changed the angle between the external magnetic field and the hard crystallographic axis of the YIG crystal. This is a well-known phenomenon related to the crystallographic anisotropy of the static magnetization of YIG crystals. For the “easy” orientation of the YIG sphere (along the [111] axis), all resonance frequencies are reduced by ~396 MHz with respect to those shown in Fig. 2.

**Figure 3:**
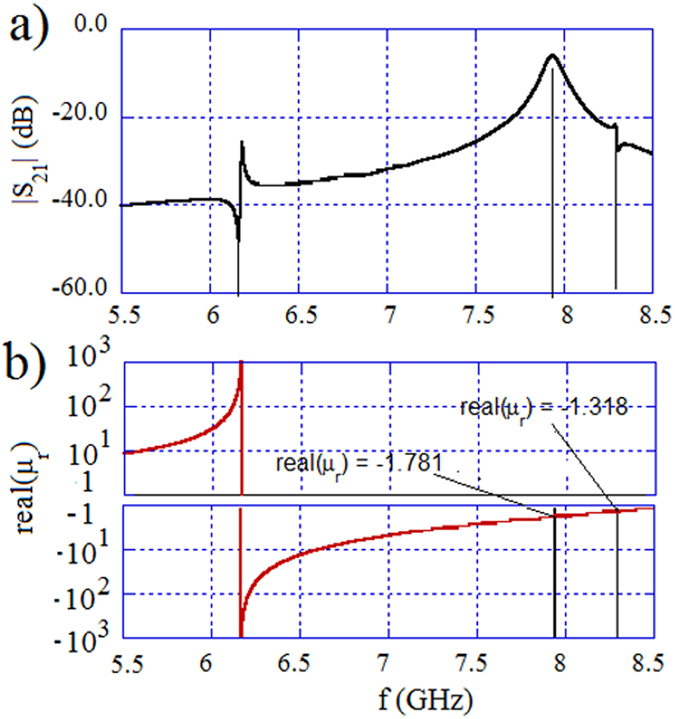
Transmittance of the YIG filter. (**a**) Transmittance of the YIG filter computed with FDTD using QW-3D. (**b**) Corresponding components of the permeability tensor obtained from Eq. (1) with the following parameters: *α* = 0.0002, *H*_0_ = 175.3 kA/m, and *M*_*s*_ = 140 kA/m.

Figure 1b depicts a simple numerical model of the filter with a YIG sphere magnetized along the z-axis. The sphere is located in a rectangular 2 × 2 x 2 mm^3^ cavity with perfectly conducting walls. We conducted an EM analysis with the FDTD method implemented in QW-3D. The model consists of about 4.1 million rectangular FDTD cells of variable sizes, with the smallest one equal to 10 μm. Figure 3a presents the resul**t**s of the FDTD computations of the transmission coefficient for the YIG filter model depicted in Fig. 1b.

The properties of the YIG sample obviously depend on the uniform static magnetic field *H*_*0*_. For a sphere, *H*_0_ is related to the external magnetic field *H*_*ext*_ by the well-known formula 
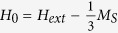
, where 1/3 is a demagnetization factor of a sphere^11^. In that case, we expect the ferromagnetic resonance frequency *f*_*res*_ for the assumed parameters of the model (listed in the caption of Fig. 3) to be ~6.166 GHz. As seen in Fig. 3a, the maximum transmission of the filter occurs at a frequency of 7.938 GHz, which is much larger than the frequency of the ferromagnetic resonance. However, it is in approximate agreement with the experimental data. As shown in Fig. 2, the main resonance occurs at 8.166 GHz for the [100] orientation of the YIG crystal. According to Fig. 3b, the filter’s transmittance at 7.938 GHz corresponds to the effective permeability for a right-handed circularly polarized wave, which is equal to *Real*(*μ*_*r*_) = *Real*(*μ* + *κ*) = −1.781. In addition to the main transmittance peak, there are two visible discontinuities in the computed transmission characteristic of the filter shown in Fig. 3a. The first discontinuity appears near the ferromagnetic resonance frequency at 6.165 GHz, where the real part of the effective permeability *μ*_*r*_ is large, while the second occurs at 8.292 GHz, where *Real*(*μ*_*r*_) = −1.318.

**Figure 4:**
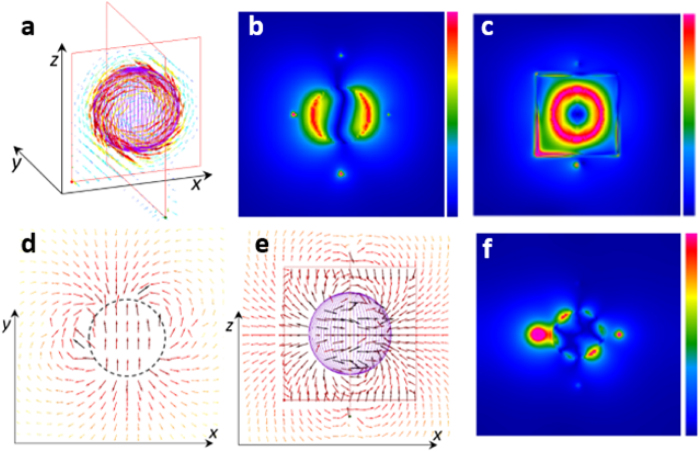
Electromagnetic field distributions in the YIG filter computed with FDTD. (**a**) Instantaneous electric field vector (7.938 GHz). (**b,c**) Magnitude of the instantaneous electric field vector (7.938 GHz). (**d,e**) Instantaneous magnetic field vector (7.938 GHz). **(f)** Magnitude of the instantaneous electric field vector (8.292 GHz). Note that the four points of field perturbation outside the YIG sample, visible in (**b**–**d**), originate from the coupling loops.

We analysed the electromagnetic field distributions to understand why the maximum transmittance of the filter occurs at a frequency that does not correspond to any characteristic features of the ferromagnetic permeability tensor. Figure 4a shows a 3D distribution of an instantaneous electric field vector at 7.938 GHz, and Fig. 4b,c present its magnitude in the *x-y* and *x-z* planes, respectively, obtained using the FDTD model shown in Fig. 1b. The electric field is circumferential and tangential to the air/YIG interface. Figures 4d,e show the corresponding 2D instantaneous magnetic field distributions in the *x-y* and *x-z* planes, respectively. The magnetic field vector in the YIG sphere has components that are almost transverse with respect to the applied static magnetic field. The polarization of the magnetic field inside the YIG sphere is reversed with respect to the magnetic field in air, due to negative real part of the permeability at the observation frequency. Figure 4f shows the magnitude of the instantaneous electric field vector at 8.292 GHz, where *real*(*μ*_*r*_) = −1.318. As for the main mode, the electric field has only a circumferential component tangential to the air/YIG interface, but there are more variations in the *x-y* plane compared to the main mode. We emphasize that the magnetic field is not evenly distributed inside the YIG sphere, but is focused at the air/YIG interface. Furthermore, the electromagnetic field rotates clockwise in time around the magnetization *z* axis, which we can observe in the time-domain simulations.

The obtained field distributions indicate that magnetic plasmons exist in the analysed system. They are equivalent to the dielectric surface plasmons at metal dielectric interfaces occurring at frequencies, where the permittivity of the metal is negative. Nano-resonances in spheres that exhibit the negative permittivity value *ε*_*r*_ ≈ −2. a called plasmonic resonances, and they have been studied by many researchers^26^,^27^,^28^,^29^,^30^. Plasmonic resonances lead to several exciting optical phenomena, including the sparkling colours of stained glasses^26^,^27^,^28^,^29^,^30^. To date, magnetic plasmon resonances have not been discovered at optical frequencies^28^ but, as it is demonstrated in this paper, they do exist at microwave frequencies.

## Resonators containing gyromagnetic medium

Hundreds of experiments with YIG spheres have been performed in different types of metal cavities, resulting in the identification and measurement of multiple resonances^1^,^2^,^3^,^4^,^5^,^6^,^7^,^8^,^9^,^13^,^14^,^15^,^16^,^17^,^18^,^19^,^20^,^21^. Most of these studies assumed that the cavity modes are coupled to the so-called quasi-magnetostatic modes (or Walker modes^5^). It seems, however, that the problem is actually more complicated. In general, EM fields in the system are solutions of the coupled Maxwell and Landau-Lifshitz-Gilbert equations with appropriate boundary conditions defined for the whole cavity containing a YIG sphere. Unfortunately, when the dimensions of the sphere are much smaller than the dimensions of the metal cavity, a numerical electromagnetic simulation becomes impractical. This is because the number of rectangular cells in the finite difference method (or the number of polyhedral elements in the finite element method) that are necessary to obtain a desired accuracy increases proportionally with the ratio of the volume of the cavity to the volume of the sphere.

For these reasons, we used analytical methods to investigate a spherical cavity loaded with a YIG sphere. It was shown earlier that YIG filters operate at a magnetic plasmon resonance, where the permeability of the gyromagnetic medium is *μ*_*r*_ ≈ −2 for rotating fields. It was also shown that, for plasmonic modes, the microwave magnetic field is orthogonal to the static magnetic field. Such modes can be rigorously analysed in an appropriately chosen coordinate system assuming that the permeability is a scalar (but dispersive) quantity, *μ*_*r*_. To analyse the eigenmodes in a YIG sphere situated at the centre of a spherical metal cavity, we chose a spherical coordinate system rotating synchronously with the circularly rotating electromagnetic fields. The rotating r-θ plane of this system corresponds to the *x-y* plane in Fig. 4, and the azimuthal coordinate of the system corresponds to the circumferential electric field. In general, TE_n0p_ and the TM_n0p_ modes exist in spherical resonators, where the n, m, and p subscripts indicate elevation, azimuthal, and radial mode orders, respectively. However, only the TE_n0p_ modes will be analysed herein, as the RF magnetic field components are orthogonal to the static magnetic field in this case, facilitating the analytical study. The resonances of the isotropic magnetic spheres for a medium with scalar complex permittivity and scalar complex permeability can be rigorously computed as solutions of the appropriate transcendental equations^31^,^32^,^33^,^34^. The equations for a sphere of radius *R*_1_ situated in free space have the following form for TE_nmp_ modes:


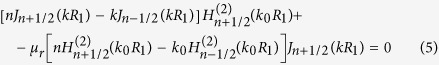


where *k* = *k*_0_(*ε*_*r*_*μ*_*r*_)^0.5^, *k*_0_ = *ω*/*c, ε*_*r*_ is the relative complex permittivity of the sphere, *μ*_*r*_ is the relative complex permeability of the sphere, *c* is the speed of EM waves in a vacuum, and *J (H*) are the Bessel (Hankel) functions.

We obtained the transcendental equation for the TE_nmp_ modes of the shielded sphere, with the radius of the shield equal to *R*_2_, by modifying the equation for the multi-layered spherical Bragg resonators^34^:


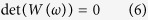


where:


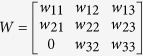



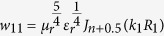



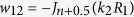



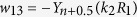



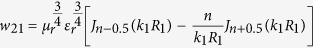



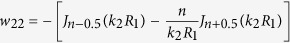



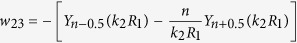



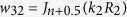



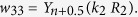


Equations (5 and 6) can be solved numerically for the complex angular frequencies. The real parts of the angular frequencies divided by 2π correspond to the resonance frequencies of particular modes. The Q-factors are determined as *Q* = Real(ω)/(2Imag(ω)). More general expressions for the EM fields in anisotropic spheres can be represented in terms of spherical harmonics^35^.

## Volume modes in gyromagnetic resonators

**Figure 5:**
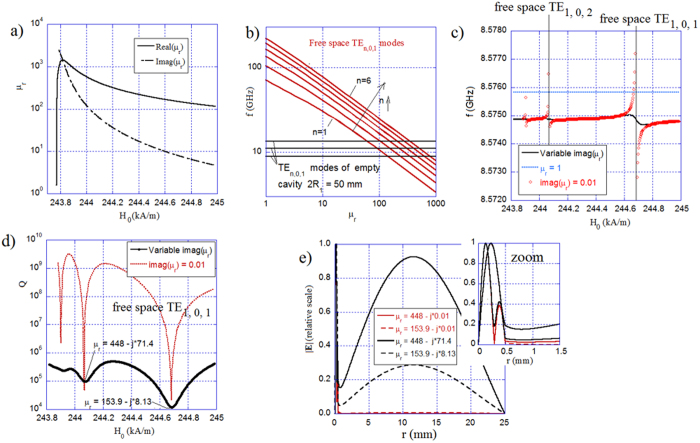
Characteristics of volume modes in the gyromagnetic sphere. (**a**) Scalar effective permeability components of a gyromagnetic medium as a function of the internal static magnetic field for *α*  = 0.0002, *M*_*S*_ = 140 kA/m, and *f*_*res*_* = *8.575 GHz. (**b**) Resonance frequencies of a few free space TE_n01_ modes of a magnetic sphere as a function of permeability. **(c)** Resonance frequencies for three TE_10p_ modes of a magnetic sphere situated in a spherical cavity versus the internal static magnetic field. The black curve corresponds to material with permeability components as in (**a**), while the red curve is for the real part as in (**a**) and *Imag*(*μ*_*r*_) = 0.01. **(d)** Q-factors for three TE_10p_ modes of the magnetic sphere versus the internal static magnetic field. **(e)** Radial distribution of the modulus of the electric field for the TE_101_ and TE_102_ modes of the magnetic sphere situated in a spherical cavity. For plots (**b**–**d**): *ε*_*r*_ = 16, *R*_1_ = 0.5 mm, and *R*_2_ = 25 mm.

For gyromagnetic samples, the magnetic losses are the largest for the internal static magnetic field value corresponding to the ferromagnetic resonance for a given frequency *f*_*res*_. As an illustration, Fig. 5a presents the scalar permeability components of a gyromagnetic medium, with *M*_*s*_ = 140 kA/m and *α* = 0.0002, for a certain range of the static magnetic fields near ferromagnetic resonance.

First, we computed a few resonance frequencies for a low loss isotropic magnetic sphere (*Imag*(*μ*_*r*_) = 0.01) situated in a free space. Relative permittivity is 16, the radius is *R*_*1*_ = 0.5 mm, and the real part of the permeability spans from 1 to 1000. The red lines in Fig. 5b represent a few of the TE_n01_ resonant free space modes of the isotropic magnetic sphere, while the black horizontal lines show the resonance frequencies of the first three TE_n01_ modes of the empty cavity with the radius of *R*_*2*_ = 25 mm. The TE_nmp_ frequencies of the empty cavity were evaluated from the well-known formula: 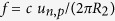, where *u*_*n*,*p*_ denotes the *p*-th root of the equation *J*_*n* + 0.5_(*u*) = 0. When the small magnetic sphere is inserted into the cavity, strong mode coupling occurs on the intersections between the free space modes of the magnetic sphere and cavity modes as indicated in Fig. 5c. As a result, the resonance frequencies (red circles in Fig. 5c) and Q-factors (red line in Fig. 5d) of the cavity vary rapidly and mode splitting occurs near the mode crossing points. The first mode crossing point appears for *Real*(*μ*_*r*_) ≈ 153.9, and the second for *Real*(*μ*_*r*_) ≈ 448. The coupling between the free space modes of the magnetic sphere and the cavity modes strongly depends on the losses of the sphere. The smaller the losses in the sample the stronger coupling takes place. The resonance frequencies and the Q-factors for a lossy sample with the imaginary part of the permeability presented in Fig. 5a are shown as black lines in Fig. 5c,d. For the sphere with large imaginary parts of permeability, the Q-factor occurring for the first mode crossing for *Real*(*μ*_*r*_) ≈ 153.9 is the most pronounced. Such behavior can be explained with an analysis of the electromagnetic field distributions in the cavity. Figure 5e presents a radial distribution of the modulus of the electric field for the TE_101_ and TE_102_ modes of the magnetic sphere situated in a spherical cavity. It can be seen that the electric field practically vanishes outside the magnetic sphere for *Imag*(*μ*_*r*_) = 0.01, while it remains substantial there for larger magnetic losses.

For this reason, we concluded that if the gyromagnetic sphere has low magnetic losses the electromagnetic energy stored in the cavity region (*R*_*1*_ < *r* < *R*_2_) is smaller than the energy stored in the sample and the total Q-factor approaches that of the sphere in free space.

## Magnetic plasmon modes in gyromagnetic resonators

As shown, magnetic plasmon modes exist for negative effective permeability. Such modes should be present in both unshielded and shielded gyromagnetic spheres. For the TE_n0p_ modes, the magnetic field has a radial component, so the magnetic plasmon modes should belong to this family of modes.

**Figure 6:**
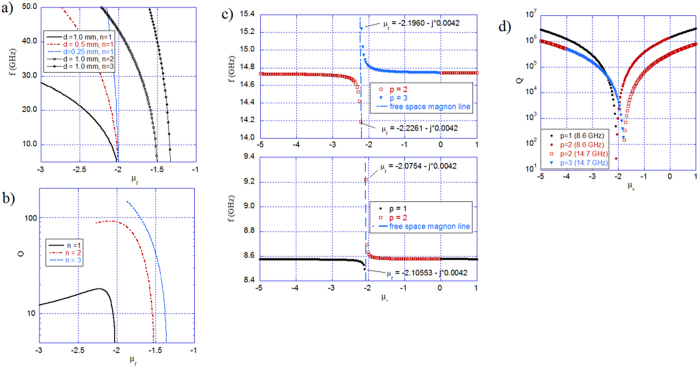
Characteristics of magnetic plasmon modes in the gyromagnetic sphere. (**a**) Resonance frequencies. **(b)** Q-factors for the free space TE_n01_ modes for magnetic spheres as a function of the real part of permeability, assuming that *Imag*(*μ*_*r*_) = 42 × 10^−4^, and *ε*_*r*_ = 16. **(c)** Resonance frequencies. **(d)** Q-factors for the TE_10p_ modes of the magnetic sphere in a spherical cavity, assuming that *Imag*(*μ*_*r*_) = 42 × 10^−4^, *ε*_*r*_ = 16, *R*_1_ = 0.5 mm, and *R*_2_ = 25 mm.

Figures 6a,b present computed resonance frequencies and Q-factors for the TE_101_ mode in free space - solutions of Eq. (5) - that are obtained for magnetic spheres of various radii having an effective permeability in the range −3 < *μ*_*r*_ < −1. In these computations, we assume that the imaginary part of the permeability is constant and equal to 42 × 10^−4^. This corresponds to the value obtained for the gyromagnetic medium, with α = 0.0002, *M*_*S*_ = 140 kA/m, and the static internal magnetic field *H*_*0*_ chosen so that *μ*_*r*_ = −2. These reproduce the conditions for the simulations presented in Fig. 3. Figure 6a shows that the resonance frequencies vary rapidly with the real part of the permeability, but at frequencies below 10 GHz they are practically the same for spheres with radii in the range 0.125 mm < *R*_*1*_ < 0.5 mm. The asymptotic permeability values at the low frequency limit for the TE_101_ mode are slightly smaller than −2 for all radii of the three spheres. These values at the low-frequency limit for the TE_201_ and TE_302_ modes for a sphere with *R*_*1*_ = 0.5 mm radius are −1.53 and −1.35, respectively.

**Figure 7:**
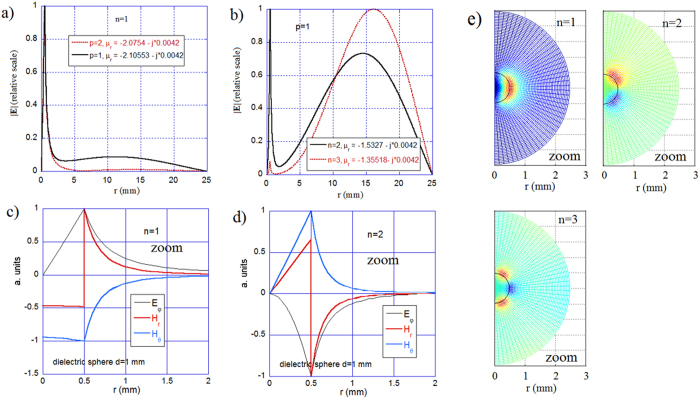
EM distributions in the spherical cavity with the magnetic sphere. (**a**) The electric field distributions for the first two magnetic plasmon TE_10p_ modes of the magnetic sphere in the spherical cavity *(R*_2_ = 25 mm). **(b)** The electric field distributions for two magnetic plasmon TE_n01_ modes of the magnetic sphere. **(c)** Radial distribution of all EM components for the TE_101_ mode in the expanded view. **(d)** Radial distribution of all EM components for the TE_201_ mode in the expanded view. **(e)** 2D plots of the electric field in the r-Θ plane for three TE_n01_ modes. For all plots: *Imag*(*μ*_*r*_) = 42 × 10^−4^, *ε*_*r*_ = 16, *R*_1_ = 0.5 mm, and *R*_2_ = 25 mm.

Next, we consider the TE_n0p_ resonances for a 0.5 mm radius gyromagnetic sphere in a 25 mm radius cavity that occur at negative scalar permeability values in the range −5 < *μ*_*r*_ < 1. Figures 6c,d present the resonance frequencies and Q-factors for the first three TE_10p_ modes, respectively. These figures show that, near the asymptotic lines corresponding to the free space magnetic plasmon modes, the resonance frequencies vary rapidly and the Q-factors approach their minima. The electric field distribution obtained for permeability values corresponding to the Q-factor minima for the modes with *p* = 1 (*μ*_*r*_ = −2.105) and *p* = 2 (*μ*_*r*_ = −2.075) are presented in Fig. 7a. The radial subscript in Fig. 7a corresponds to the first magnetic plasmon mode in the sphere. However, for the mode with *p* = 2, the electric field changes its sign in the air region, and for the mode with *p* = 1, the electric field is evanescent in the air region without any change in its sign.

Magnetic plasmon mode resonances are also observed for higher mode indices (*n* ≥ 1), but their coupling with the cavity modes decreases with an increase in the mode index. The electric field distributions for the modes with *n* = 2 and *n* = 3 are presented in Fig. 7b for such permeability values, as they permit the strongest coupling. Figures 7c,d present the radial distributions for all three electromagnetic field components for the modes with *n* = 1 and *n* = 2 in an expanded view that includes the gyromagnetic sphere. The magnetic field components are finite at the centre of the sphere for the TE_101_ mode, and the same is true for the TE_10p_ modes. For the modes having elevation indices *n* ≥ 1, the magnetic field components approach zero at the centre of the sphere. For all of the TE_n0p_ modes, the electric field vanishes at the centre of the sphere. Figure 7e presents distributions of the electric field in the r-Θ plane for the TE_n01_ modes, which are seen to be similar to the distributions shown in Fig. 4b,d. This indicates that the mode presented in Fig. 4d is the magnetic plasmon mode TE_301_.

## Discussion

This work shows that maximum transmission of YIG filters corresponds to the frequency of the first magnetic plasmon resonance and not to the ferromagnetic resonance, which plays only supplementary role in bringing negative effective permeability at a discrete frequency. We rigorously analysed the resonances for a gyromagnetic sphere in a free space and in a spherical cavity. Both magnetic plasmon modes (modes that appear for the negative effective permeability of a gyromagnetic medium) and volume modes (modes that appear for a large positive permeability) were analysed. Results show that the strongest coupling between the free space modes and the modes of the empty cavity occur near the free space and cavity mode crossing points. Another interesting observation is that the fundamental mode of operation of the YIG filter is not circularly polarized but it circularly rotates in time around the magnetization axis, which allows coupling between orthogonally oriented coupling loops.

The internal static magnetic field values corresponding to the mode crossing points for the magnetic plasmon modes differ from the values corresponding to the ferromagnetic resonance. If we assume that the magnetic losses are negligibly small (*α* ≈ 0), Eqs (2 and 3) can be used to derive the following:


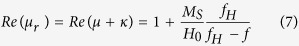


Substituting *f*_*H*_ = *γH*_*0*_ into Eq. (7), we find that the internal static magnetic field *H*_*μp*_, which is necessary to obtain a fixed *μ*_*p*_ value of the scalar permeability, is equal to


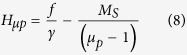


It is interesting to note that the internal static magnetic value of *H*_*μp*_, which is necessary to obtain the scalar permeability *μ*_*p*_ = −2, is equal to 
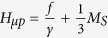
. When the influence of anisotropy is neglected, 
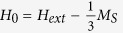
, so the first magnetic plasmon mode should be observed near the external static magnetic field 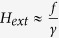. Equality in this formula would take place only for a specific orientation of YIG crystal with respect to the static magnetic field.

## Additional Information

**How to cite this article**: Krupka, J. *et al*. Electrodynamic study of YIG filters and resonators. *Sci. Rep.* **6**, 34739; doi: 10.1038/srep34739 (2016).

## Supplementary Material

Supplementary Dataset 1

Supplementary Dataset 2

Supplementary Dataset 3
